# Screening for social determinants of health among populations at risk for MASLD: a scoping review

**DOI:** 10.3389/fpubh.2024.1332870

**Published:** 2024-04-10

**Authors:** Rebecca G. Kim, April Ballantyne, Molly B. Conroy, Jennifer C. Price, John M. Inadomi

**Affiliations:** ^1^Division of Gastroenterology and Hepatology, Department of Internal Medicine, University of Utah, Salt Lake City, UT, United States; ^2^Division of General Internal Medicine, Department of Internal Medicine, University of Utah, Salt Lake City, UT, United States; ^3^Division of Gastroenterology and Hepatology, Department of Medicine, University of California San Francisco, San Francisco, CA, United States; ^4^Department of Internal Medicine, School of Medicine, University of Utah, Salt Lake City, UT, United States

**Keywords:** metabolic dysfunction-associated steatotic liver disease, SDoH screening, obesity, diabetes, hypertension

## Abstract

**Background:**

Social determinants of health (SDoH) have been associated with disparate outcomes among those with metabolic dysfunction-associated steatotic liver disease (MASLD) and its risk factors. To address SDoH among this population, real-time SDoH screening in clinical settings is required, yet optimal screening methods are unclear. We performed a scoping review to describe the current literature on SDoH screening conducted in the clinical setting among individuals with MASLD and MASLD risk factors.

**Methods:**

Through a systematic literature search of MEDLINE, Embase, and CINAHL Complete databases through 7/2023, we identified studies with clinic-based SDoH screening among individuals with or at risk for MASLD that reported pertinent clinical outcomes including change in MASLD risk factors like diabetes and hypertension.

**Results:**

Ten studies (8 manuscripts, 2 abstracts) met inclusion criteria involving 148,151 patients: 89,408 with diabetes and 25,539 with hypertension. Screening was primarily completed in primary care clinics, and a variety of screening tools were used. The most commonly collected SDoH were financial stability, healthcare access, food insecurity and transportation. Associations between clinical outcomes and SDoH varied; overall, higher SDoH burden was associated with poorer outcomes including elevated blood pressure and hemoglobin A1c.

**Conclusion:**

Despite numerous epidemiologic studies showing associations between clinical outcomes and SDoH, and guidelines recommending SDoH screening, few studies describe in-clinic SDoH screening among individuals with MASLD risk factors and none among patients with MASLD. Future research should prioritize real-time, comprehensive assessments of SDoH, particularly among patients at risk for and with MASLD, to mitigate disease progression and reduce MASLD health disparities.

## Introduction

**Figure 1:**
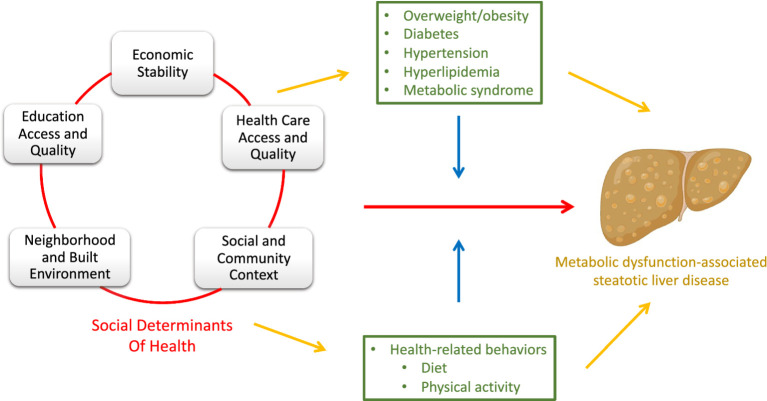
Simplified directed acyclic graph (DAG) depicting the relationship between social determinants of health (SDoH) and metabolic dysfunction-associated steatotic liver disease (MASLD). The DAG includes the direct effect of SDoH on MASLD (red pathway), as well as the indirect effect through mediators (yellow pathway). The blue pathway represents the way in which the covariates modify the effect of SDoH on MASLD (effect modifiers). Clinical conditions related to MASLD, and health-related behaviors are green representing their roles as both mediators (yellow) and effect modifiers (blue). *Made with graphics from Biorender.com.

Metabolic-dysfunction associated steatotic liver disease (MASLD), the most common chronic liver disease in the United States, (1) disproportionately impacts vulnerable populations including non-White racial/ethnic groups and individuals with lower income and education (2–5). MASLD is strongly associated with obesity, diabetes, hypertension, dyslipidemia and metabolic syndrome (6–8). In fact, it is the hepatic manifestation of metabolic syndrome; the development of liver inflammation and fibrosis in MASLD is a result of nutrition, insulin resistance, and lipotoxicity (6, 9). Metabolic syndrome and its individual components are also more prevalent among vulnerable populations (10–16). There is a growing body of evidence that health disparities including those observed in chronic liver disease, are primarily the result of social determinants of health (SDoH) (17–23), the conditions where people are born, live, learn and work (24). Informative studies include systematic reviews and meta-analyses that show the associations of low socioeconomic status with (1) increased obesity prevalence (15), (2) higher hemoglobin A1c among individuals with diabetes (11), and (3) higher diabetes-related mortality (25). Data from the Centers for Disease Control and Prevention show higher prevalence of diabetes among non-White racial/ethnic groups; (10) based on national data, obesity is associated with lower household income and lower education level (16). Although the direct relationship between SDoH and MASLD is less well-studied, emerging evidence has shown that education (5) and race/ethnicity (4) are associated with MASLD prevalence and severity, neighborhood-level SDoH is associated with mortality, complications and cardiovascular disease, (26) and food insecurity is associated with increased risk of advanced liver fibrosis and all-cause mortality among those with MASLD (Figure 1) (2, 24, 27–30).

Based on these data, as well decades of other studies with similar findings, (31–35) societies have recommended SDoH screening in clinical practice (36–38). In the Standards of Care in Diabetes guideline published in 2023, incorporation of SDoH into patient care is recommended to improve diabetes care, specifically for individualized self-management of diabetes and when selecting pharmacologic agents (36). In the most recent American Academy of Family Physicians’ guidelines for hypertension, providers are recommended to screen for SDoH and be conscious of how SDoH impact patient care, and the guidelines state future research assessing the impact of SDoH should be prioritized (37). The 2023 guidelines for Management of Patients with Chronic Coronary Disease developed by multiple cardiology societies recommend “routine assessment by clinicians and the care team for SDoH to inform patient-centered treatment decisions” (38). Although hepatology guidelines do not explicitly recommend SDoH screening, several recent reviews and editorials in top hepatology and liver transplant journals have concluded that SDoH data must be collected systematically to address liver disease disparities and improve patient outcomes overall (3, 39–41).

Despite these recommendations to screen for SDoH, a consensus on how to successfully conduct SDoH screening is lacking and a systematic approach for broad use has not been developed (42). The purpose of this scoping review is to describe if and how SDoH screening is being conducted among patients with MASLD and those at highest risk of MASLD. We aimed to summarize current efforts to screen for and address SDoH within hepatology clinics, primary care clinics, and other outpatient and inpatient settings, specifically among individuals with MASLD and its risk factors.

## Materials and methods

We performed a scoping review, which uses a systematic search to bring together literature covering topics with emerging evidence (43, 44). Through a summarization of the body of literature addressing our research question, we aimed to (1) report on current evidence that addresses and informs practice and (2) identify gaps in the research knowledge. We hypothesized that current literature would contain a breadth of studies describing in-clinic SDoH screening for patients with MASLD risk factors, but there would be few, if any, studies among those with MASLD.

### Search strategy

The literature search was completed just following the multi-society announcement of the change in nomenclature for non-alcoholic fatty liver disease (NAFLD) to MASLD on June 24, 2023 (45). Therefore, as all studies published up until that point referred to patients with MASLD as NAFLD, our search was conducted using NAFLD and terms with “fatty liver disease” instead of steatotic liver disease.

To identify all relevant articles that describe screening for SDoH among adults with MASLD-related risk factors, we conducted a systematic literature search of MEDLINE (1946 through July 2023), Embase (1988 through July 2023), and CINAHL Complete (1937 through July 2023) databases, with no language restrictions. The search strategy was designed after consultation with a librarian and implemented by the study’s investigators using the search strategy as described in the Supplementary Tables. The search for SDoH was designed using (1) different phrases similar to SDoH like “socioeconomic determinant” and “health structural determinant,” and (2) categories of SDoH as defined by Healthy People 2030, including “economic stability,” “education access,” “health care quality,” etc. (24). Two reviewers (RGK, AB) independently assessed the title and abstract of studies identified in the primary search for inclusion, and the full text of remaining articles were examined to determine whether they met inclusion criteria (46). Bibliographies from the selected articles and review articles on the topic were manually searched for additional studies. Any conflicting decisions were reviewed by RGK and discussed with co-authors as needed.

### Study selection

Studies were included if they met the following inclusion criteria: (1) conducted in adults with or at risk for overweight/obesity, diabetes, hypertension, hyperlipidemia or MASLD, (2) collected SDoH data from screening performed in a clinical setting defined as real-time SDoH screening associated with a clinical encounter, and (3) assessed the prevalence or association of SDoH with clinical outcomes including incidence or disease severity. We excluded international and non-English studies, those reporting health-related behaviors (e.g., physical activity, dietary choices, etc. which are impacted by SDoH but are not considered to be SDoH) or built environment, intervention studies, perceived health or quality of life as their clinical outcome, and epidemiologic studies, particularly those conducting retrospective analyses of large national databases.

### Data extraction and analysis

Data collected from each study included the following: time period of the study, location, patient population, SDoH screening questions used, setting of screening, clinical outcomes reported, and association of SDoH and clinical outcomes of interest including overweight/obesity, diabetes, hypertension, hyperlipidemia or MASLD.

## Results

### Search results

**Figure 2:**
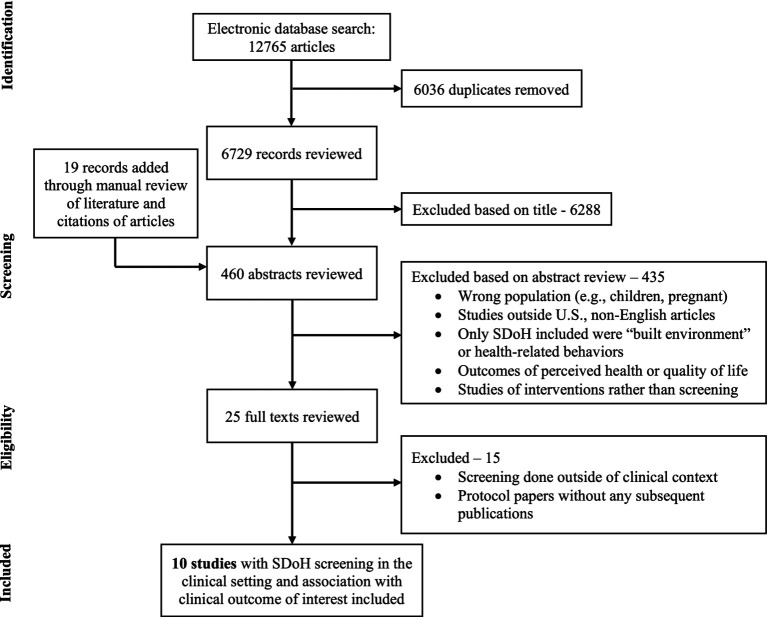
Flow diagram of study selection.

Of the 6,729 unique studies identified using our search criteria, after reviewing 460 abstracts and 25 full texts, 10 studies met our inclusion criteria – 8 manuscripts and 2 conference abstracts (47–56). Figure 2 shows the flow diagram summarizing our study identification and selection. Notably, there were no studies identified among individuals with MASLD.

### Characteristics of included studies

The characteristics of these studies are shown in Table 1. Overall, these studies included 148,151 participants, of whom 89,408 (60%) had diabetes and 25,539 (17%) had hypertension. All studies were conducted in primary care clinics; 7 were specifically done in clinics affiliated with an academic medical center, one was performed in community health centers, and another was completed in federally qualified community health center (FQHC) adult and family medicine clinics. Two studies were limited to patients with hypertension and three studies only included individuals with diabetes. The other five studies included all primary care patients, but specifically assessed for the association of SDoH with overweight/obesity or body mass index (BMI), hypertension, diabetes, and/or atherosclerotic cardiovascular disease (ASCVD) risk.

**Table 1:** Characteristics of included studies describing social determinants of health screening in a clinical setting to populations at risk for metabolic-dysfunction associated steatotic liver disease.

Study	Location	Time Period	Baseline patient population	Number in study	Screener used	Setting of screening	Reported clinical outcomes	Association between SDoH and clinical outcomes observed
Arunthamakun et al., 2023 (47)	Pittsburgh, PA	2019–2022	adults with hypertension seen in primary care	6,131	Not specified	two internal medicine outpatient residency clinics	uncontrolled hypertension	Increased instances of uncontrolled hypertension in Black populations; Black populations also reported higher prevalence of food insecurity, financial insecurity, environmental risk, poor healthcare education
Berkowitz et al., 2016 (48)	Boston, MA	October 2013 – April 2014	adults seen in primary care	3,166	Health Leads program screener	academic, hospital-based primary care practices with care-management programs for high-risk Medicare patients	diabetes, hypertension	Patients reporting social needs (*N* = 416) were more likely to be women, racial/ethnic minorities, and have Medicaid insurance. The prevalence of diabetes and hypertension was higher among those with unmet social needs. Diabetes (aOR 1.7), poorly controlled diabetes (aOR 1.83) and hypertension (aOR 1.83) were all associated with food insecurity.
Brady et al., 2021 (49)	Kansas City, KS	October 2017 – September 2018	adults seen in primary care; excluded if pregnant or diagnosed with type I diabetes	26,093	modified Health Leads program screener	primary care clinics affiliated with academic healthcare system	diabetes	Among individuals who reported at least one social need, the odds of having diabetes was 1.7 times higher than among those without social needs. All social needs were more commonly reported in patients with diabetes, except for need for childcare services. The social needs with the highest odds were prescription cost (OR 2.0), transportation (OR 1.9), and health literacy (OR 1.8).
Chambers et al., 2021 (50)	New York City, NY	April 2018 – December 2019	adults with diabetes seen in primary care	5846*	10-item screener adapted from Health Leads screener	primary care clinics affiliated with academic medical center	controlled vs. uncontrolled diabetes, (hemoglobin A1c ≥ 9)	Among patients with diabetes, those reporting unmet social needs were more likely to have uncontrolled diabetes. On multivariable analysis, the number of reported social needs (1, 2, ≥3) was associated with greater odds of uncontrolled diabetes in a dose–response relationship, (aOR uncontrolled diabetes, aOR 1.19 for 1 social need, aOR 1.36 2 social needs, aOR 1.59 ≥ 3 social needs). Housing insecurity, food insecurity and healthcare transportation were all associated with higher odds of uncontrolled diabetes.
Drake et al., 2021 (51)	Durham, NC	May 2017–February 2019	adults seen in federally qualified community health center (FQHC) Adult Medicine and Family Medicine Clinics	2,192	PRAPARE	FQHC Adult and Family Medicine clinics	body mass index, systolic and diastolic blood pressures, and ASCVD 10-year risk	In the development of predictive models for clinical outcomes, social factors improved c-statistic. (1) Obesity -unemployment, housing instability, transportation instability, stress; (2) hypertension -members per household, migrant/seasonal work, unemployment, uninsured, access to health care; (3) ASCVD risk -uninsured, unemployment, housing instability, low social interaction, stress, fees unsafe in residence, food insecurity, access to health care, childcare, lack of phone
Gold et al., 2022 (52)	Pacific Northwest	July 2016 – February 2020	adults with diabetes seen in primary care	73,484	Not specified	community health centers	type 2 diabetes clinical care and diabetes clinical outcomes including A1c <9%, blood pressure and cholesterol	Among individuals with diabetes who were up-to-date with their clinical care, housing insecurity was not associated with any clinical outcomes. Food insecurity was associated with lower prevalence of controlled A1c. Transportation insecurity was associated with lower rates of (1) controlled A1c, (2) blood pressure at goal and (3) LDL at goal
Heller et al., 2021 (53)	New York City, NY	April 2018 – December 2019	adults seen in primary care clinics	33,550	10-item screener adapted from Health Leads screener	primary care clinics affiliated with academic medical center	chronic medical conditions including hypertension, obesity, and diabetes	Assessed the association between number of social needs reported and health outcomes including hypertension, obesity, and diabetes. For each outcome, the adjusted prevalence ratio increased with higher number of reported social needs. Healthcare transportation insecurity was the most strongly associated with most clinical outcomes. Inability to cover healthcare costs was associated with 1.13 prevalence ratio of obesity.
Okoh et al., 2020 (54)	Boston, MA	May 2019 – July 2019	adults with hypertension	86	PI-FENCES model	ambulatory clinic, inpatient, seen by internal medicine residents	hypertension	47% of hypertensive patients seen in clinic/or inpatient were uninsured; hypertensive patients without insurance were more likely to be admitted to the inpatient service or ICU
Palacio et al., 2020 (55)	Miami, FL	September 2016 – September 2017	adults seen in primary care	2,229	National Academy of Medicine Committee Social and Behavioral Domains	primary care clinics affiliated with academic medical center	Framingham risk score for cardiovascular disease, modifiable CVD risk factors -blood pressure, LDL, BMI, tobacco use, stress, A1c (w/diabetes)	Using a weighted SDoH score (developed using confirmatory factor analysis), found worse SDoH score was associated with increasing cardiovascular risk
Roth et al., 2023 (56)	Portland, OR	August 2019 –November 2020	patients with diabetes identified as having care gaps including overdue for A1c test, foot exam, eye exam	1,220	food insecurity, PRAPARE, utilities and financial security	primary care clinics, within the Providence Diabetes Collective Impact Initiative –a diabetes treatment intervention	systolic blood pressure, diastolic blood pressure, hemoglobin A1c; referral to diabetes educator, referral to community resource desk	Among the treatment group with a higher proportion of individuals undergoing SDoH screening (26.1%), there was no difference in blood pressure or hemoglobin A1c; did not look at specific, individual SDoH and clinical outcomes

*This is a subset of Heller et al. (patient numbers not added to totals).

PRAPARE, Protocol for Responding to and Assessing Patients’ Assets, Risks, and Experiences; ASCVD, atherosclerotic cardiovascular disease; CVD, cardiovascular disease; BMI, body mass index; PCP, primary care provider; RR, relative risk; PI-FENCES, Provider, Insurance; Food, Economic stability, Neighborhood, Culture and Language, Education and Social support; SDoH, social determinants of health.

In nine of the 10 studies, only participants who completed SDoH screening were including in analyses. The number eligible for screening was not reported in most, however Palacio et al. reported an SDoH screening response rate of 36% among patients scheduled in primary care clinic (55). In the tenth study by Roth et al., participants were randomized to standard of care primary care clinics or to those included in the Diabetes Collective Impact Initiative. SDoH screening was available in either setting as it was embedded into existing clinical workflows, however remained less than 30% overall. Screening rates were higher among clinics within the Diabetes Collective Impact Initiative, where 26.1% of patients were screened, compared to standard of care primary care clinics with 1.5% screened (56). The study did not describe barriers to SDoH screening or specific differences between clinics in their approaches to SDoH screening.

### Methods for SDoH screening

Based on the information provided, each study used different SDoH questions; however, some did use previously developed and validated questionnaires. Four studies used a version of the SDoH screener used in the Health Leads program (48–50, 53). Two studies used the Protocol for Responding to and Assessing Patients’ Assets, Risks, and Experiences (PRAPARE) screening tool (51, 56, 57). One study supplemented this with the validated 2-question screener for food insecurity (58). One study described using the PI-FENCES model, which includes Provider, Insurance, Food, Economic stability, Neighborhood, Culture and Language, Education and Social support, (54) while another asked similar questions based on the National Academy of Medicine Committee Social and Behavioral Domains (59, 60).

Among the studies that included specifics regarding survey administration, the most common approaches were collection via patient interview by the provider (51, 54) or clinic staff; in Palacio et al., patients were sent messages via text or email asking them to access their patient portal to complete the SDoH survey. The two remaining studies described collection of SDoH in clinic but did not provide additional details. In general, details regarding screening methodology and implementation of SDoH screening were limited.

### Specific SDoH collected

The social determinants collected in each study are summarized in Table 2. There was not a single SDoH factor that was asked in all studies. Most (9/10) studies asked participants about financial stability (income, ability to pay utilities, afford childcare, afford medications, etc.) and food insecurity. Eight of the ten studies asked about transportation insecurity and healthcare access (primary care provider, regular care or delay in care in last 12 months, etc.). Other commonly collected SDoH (among 5–6 studies) included social isolation (participation with groups, talking with friends/family, feeling lonely, etc.), neighborhood and stress.

**Table 2:** Specific social determinants of health included in each study.

	Language	Education	Income or financial stability	Employment	Insurance	Food insecurity	Transportation	Housing stability	Social isolation	Healthcare access	Neighborhood	Stress	Other*
Arunthamakun			x			x	x		x		x	x	x
Berkowitz			x	x		x	x	x		x			x
Brady			x			x	x		x	x	x		x
Chambers			x			x	x	x		x			x
Drake	x	x	x	x	x	x	x	x	x	x	x	x	x
Gold						x	x	x					
Heller			x			x	x	x		x			x
Okoh	x	x	x		x	x			x	x	x		x
Palacio		x	x						x	x		x	x
Roth	x	x	x	x	x	x	x	x	x	x	x	x	x

^*^Birthplace (Palacio), health literacy (Arunthamakun, Brady, Palacio), veteran status & migrant/seasonal farm work (Drake, Roth), culture (Okoh), housing quality (Brady, Chambers, Heller), interpersonal violence (Chambers, Heller), legal help (Berkowitz, Chambers, Heller).

### Prevalence of SDoH

Only some studies included specific values for SDoH prevalence. Drake and colleagues collected SDoH in FQHC adult and family medicine clinics, starting in 2017. Among the 2,192 participants, 58.4% were uninsured, 34.6% obtained less than a high school education, 29% were unemployed and not seeking work, 19.4% were without housing and 16.2% reported food insecurity. Additional values can be found in the study (51). Palacio and colleagues reported the following among their study population: 39% were born outside the US, 21% had less than a high school education, 9% reported health illiteracy, 33% had financial strain, 22% described “quite a lot or very much stress,” a range from 9 to 56% responded yes to questions indicative of social isolation, 2% of participants’ income was at or below the federal poverty level and 31% reported a delay in medical care in the prior 12 months (55). In Berkowitz et al., of the 3,166 participants, 416 reported at least one social need. The prevalence of social needs ranged from 5.9% of people needing legal assistance up to 40.1% reporting food insecurity and 46.5% with healthcare needs including difficulties with insurance coverage and affording medications (48).

### Use of SDoH data

Among the 10 studies reviewed, four of the studies described clear action plans for the SDoH data collected from patients. Drake et al. referred patients to community resources or social services based on their identified needs (51). Similarly, Roth et al. referred individuals to their community resource desk staffed by multilingual and multicultural resource specialists employed by community partners to assist with the navigation of community services including nutrition assistance, housing and employment support, and dental care. When appropriate, patients were also enrolled in their Diabetic Transportation Program (56). Berkowitz and colleagues also connected participants with social needs to community resources. In their study, participants with defined social needs worked alongside an advocate who linked them to appropriate resources based on eligibility, desirability and accessibility. The study included details of their resource connections: successful referrals were most frequent for health-related needs like prescription assistance (35% of cases) and adult health insurance (15%), referrals to food pantries and soup kitchens (56%), and utilities like electric, gas and oil discount rates (49%) (48).

Okoh et al. described future directions for use of SDoH data that included the development of a follow up and referral plan for uninsured patients presenting to the inpatient setting, as well as free health screening for families and friends of uninsured individuals, components of their Reducing ReAdmission Secondary to Hypertension proposal (54).

### Associations of specific SDoH with clinical outcomes

A variety of outcomes were reported (Table 1). In general, SDoH indicative of increased social risk or social needs were associated with poorer clinical outcomes. Arunthamakun et al. found that Black populations with higher prevalence of hypertension had concurrent increased rates of household-level economic and social disparities (47). Additionally, patients with hypertension without insurance, based on findings by Okoh et al., were more likely to be admitted inpatient or to the intensive care unit likely as a result of limited healthcare access and associated financial strain (54).

Drake et al. reported the prevalence of each SDoH factor among their FQHC population overall as described above, but also specifically among those with (1) obesity, (2) elevated blood pressure, and (3) increased ASCVD risk. They did not observe differences in all SDoH, (e.g., uninsured, less than high school education), however, for the following, a higher prevalence of social risk or social need was identified among those with metabolic dysfunction and increased cardiovascular risk. Overall, 29% were unemployed not seeking work, which was 29% among obese individuals, 33.9% with elevated blood pressure, and 39.3% with high ASCVD risk. Nineteen percent were unhoused, which was 16.6% among those with obesity, 21.4% with elevated blood pressure, and 21.6% in those with high ASCVD risk. Food insecurity overall was 16.2%, increased to 16.4, 20, and 20.4% among those with obesity, high blood pressure, and high ASCVD risk, respectively (51).

Berkowitz et al. and Brady et al. described a higher prevalence of diabetes and hypertension among people with unmet social needs (48, 49). Moreover, Chambers et al. and Gold et al. found that, among patients with diabetes, social needs were associated with poorer control of diabetes based on higher hemoglobin A1c levels (50, 52).

## Discussion

In this scoping review of 10 studies on SDoH screening in the clinical setting among 148,151 patients at-risk for MASLD, we made several key observations. First, despite strong evidence and general acceptance that SDoH powerfully impact health outcomes, including MASLD risk factors and MASLD related complications, there are few studies describing real-time SDoH screening in the clinical setting, a critical gap in the current literature. Second, among studies with SDoH screening, specific SDoH factors as well as a higher burden of SDoH were associated with poorer clinical outcomes including (1) higher prevalence of diabetes and hypertension, (2) poorly-controlled hypertension, (3) higher hemoglobin A1c among individuals with diabetes, and (4) higher ASCVD risk score. Third, there was little consistency in the SDoH screening methods; a variety of questions were asked and the specific SDoH factors collected differed across studies. Fourth, only 4 of the included studies described how SDoH data were used; 3 studies connected those with social needs to available community resources.

Numerous retrospective, epidemiologic studies have shown an association between SDoH and overweight/obesity, diabetes, hypertension and hyperlipidemia (61–66). These studies often use national databases that collect data on social factors via surveys or link census data by geographic location and use area or neighborhood deprivation indices. They have described associations between race/ethnicity, education, income, type of insurance, and food insecurity (among several other SDoH) with self-reported medical conditions or international classification of diagnoses codes (2, 11–16, 31, 62, 65, 66). Several studies have been published using various large databases like the National Health and Nutrition Examination Survey, the National Health Interview Survey, and the Behavioral Risk Factor Surveillance System, etc. Specifically, through our literature review, we identified over 110 studies that used data from more than 25 unique epidemiologic databases to establish the link between SDoH and chronic diseases.

Now that these relationships are established, the next step should be to act on these findings in real-time; however, the optimal method to systematically gather SDoH information in the clinical setting is unknown. While our scoping review included studies with SDoH screening in the clinical setting and clinical outcomes, De Marchis and colleagues recently reviewed implementation science studies describing SDoH screening in the clinical setting (42). In their systematic scoping review, they summarized 41 studies describing the implementation of SDoH screening in the clinical setting. They identified steps necessary for success, described remaining challenges of real-time SDoH screening, and defined ongoing critical gaps including the need for practices that maximize screening reach, adoption, and sustainability in clinical settings (42). As more healthcare systems incorporate SDoH screening into clinical practice, the standardization of SDoH screening is critical for collection of comparable and generalizable data. More specifically, in hepatology practice, systematic SDoH screening among patients with MASLD can serve as an example for screening among individuals with other chronic liver diseases and may also be implemented in the transplant setting where disparities are known to be prevalent (67).

**Figure 3:**
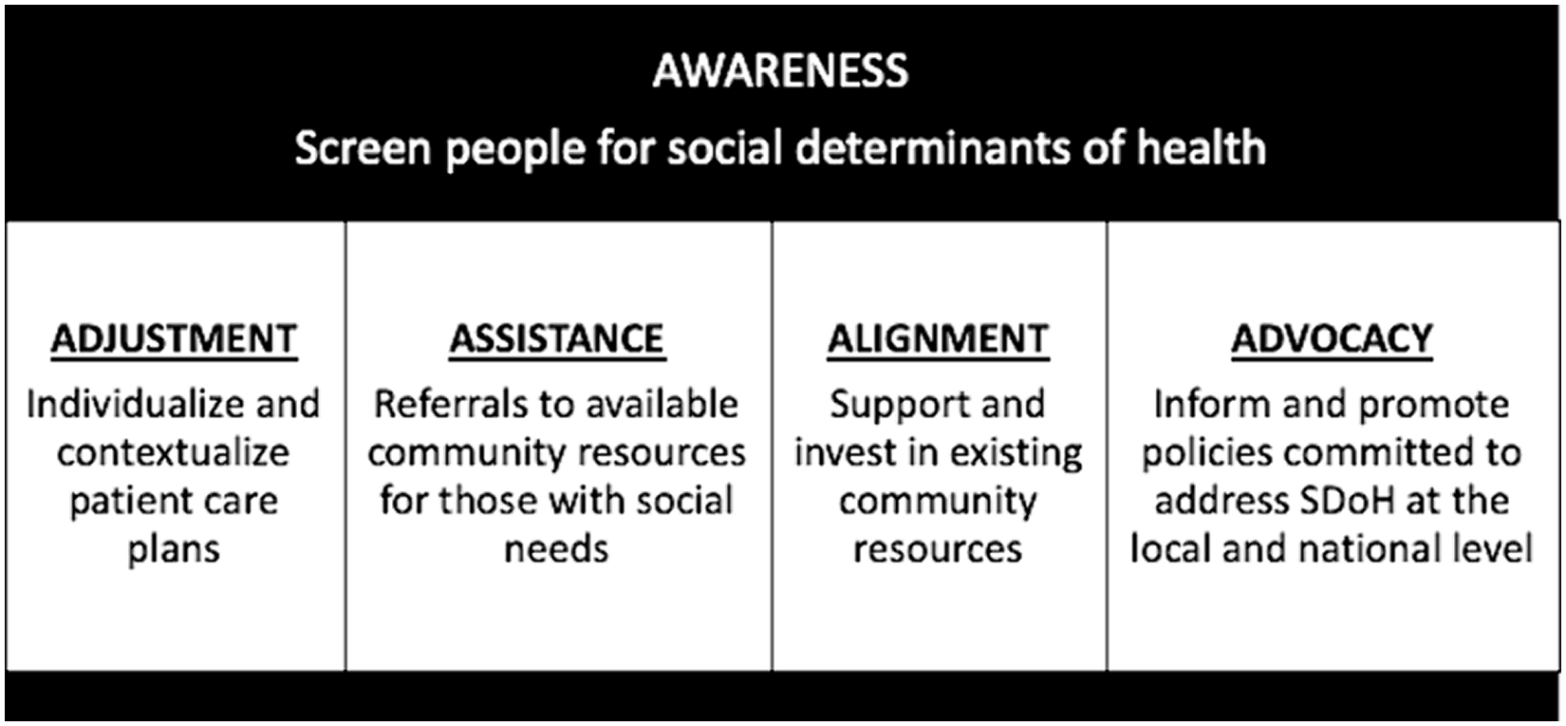
Proposed long-term health systems outcomes that result from standardized, comprehensive social determinants of health screening – Awareness, Adjustment, Assistance, Alignment, and Advocacy.

Potential next steps include (1) optimization of a single screening tool that can be systematically incorporated into clinical flow among healthcare systems nationally, (2) strategies to incentivize clinicians and healthcare systems to conduct SDoH screening regularly, and (3) normalization of SDoH screening with the general population to improve acceptability. Historically, barriers to collect SDoH data in the clinical setting included time limitations, discomfort from providers and/or patients, and the absence of solutions or resources when social needs are identified (68–72). Among the 10 studies reviewed, 6 studies incorporated SDoH data into the electronic medical record making it easily collected and readily available. Three of the studies included clear action items for providers when social needs were identified. While useful, overall data were limited and insufficient to guide the standardization of SDoH screening among individuals with or at risk for MASLD. In the future, with successful implementation of standardized, sustainable SDoH screening, (1) patients will benefit from individualized and contextualized care plans, (2) social needs may be addressed through referrals to available community resources, (3) higher utilization of community-based resources will demonstrate their value potentially leading to greater financial support and the development of similar interventions, and (4) data collected may inform and promote health policies to address SDoH at the local and national level, (Figure 3) (73).

A strength of this scoping review is the comprehensive and systematic literature search with well-defined inclusion criteria. The limitations of the study include the results of our literature search and the small number of studies that met our inclusion criteria. Only 10 studies were identified, and 2 were only published as conference abstracts with limited data available. This demonstrates an important gap in the literature. Another limitation is the heterogeneity of SDoH screening methods; different screening tools were used that collected different determinants and different approaches were used to administer surveys. Moreover, only 3 studies described what providers did when SDoH were identified. Although we hoped that a review of the literature could inform specific guidelines for how to screen for SDoH, instead it demonstrated the paucity of studies and the work that remains to be done.

In conclusion, prospective SDoH screening in the clinical setting adds to the existing data that SDoH are associated with disparate health outcomes, particularly related to overweight/obesity, diabetes, and hypertension. Real-time screening allows for incorporation of SDoH into patient care, specifically among patients with MASLD and its risk factors. However, barriers to SDoH screening remain, including the lack of consensus regarding which standardized screening tool to use and optimal approaches to achieve feasible, acceptable and sustainable SDoH screening in patient care settings. Future studies should be designed to effectively incorporate standardized SDoH screening into clinical practice to examine specific SDoH and define impactful determinants among individuals with MASLD. These data, along with input from patients, staff and communities, can then be used to develop and implement effective SDoH interventions to improve clinical care and reduce health disparities observed among populations with MASLD.

## Data availability statement

The original contributions presented in the study are included in the article/Supplementary material, further inquiries can be directed to the corresponding author.

## Author contributions

RK: Conceptualization, Data curation, Investigation, Methodology, Writing – original draft, Writing – review & editing. AB: Data curation, Writing – review & editing. MC: Methodology, Supervision, Writing – review & editing. JP: Methodology, Supervision, Writing – review & editing. JI: Methodology, Supervision, Writing – review & editing.
