# Supplementary material for: Screening for social determinants of health among populations at risk for MASLD: a scoping review
Source: Front Public Health. 2024 Apr 10;12:1332870. doi: 10.3389/fpubh.2024.1332870 (PMC11041393; doi:10.3389/fpubh.2024.1332870)
Supplement: Supplementary file 1 [file Table_1.docx]

**Supplemental Tables. Literature search strategy.**

**Medline:**

NAFLD:

| (((((("social determinants of health"[mesh] or "social risk"[tiab] or "social determinant"[tiab] or "social determinants"[tiab] or "socioeconomic determinant"[tiab] or "socioeconomic determinants"[tiab] or "health determinant"[tiab] or "socioeconomic factor"[tiab] or "socioeconomic factors"[tiab] or "health determinants"[tiab] or "health structural determinant"[tiab] or "health structural determinants"[tiab] or "economic stability"[mesh] or "economic stability"[tiab] or "education access and quality"[mesh] or "education access"[tiab] or "education quality"[tiab] or "health care access and quality"[mesh] or "health care access"[tiab] or "healthcare access"[tiab] or "health care quality"[tiab] or "healthcare quality"[tiab] or "neighborhood and built environment"[mesh] or "neighborhood environment"[tiab] or "built environment"[tiab] or "social and community context"[mesh] or "social context"[tiab] or "community context"[tiab])) and ("mass screening"[mesh:noexp] or "multiphasic screening"[mesh] or "health surveys"[mesh:noexp] or "screen*"[tiab] or "questionnaire"[tiab] or "questionnaires"[tiab] or "survey"[tiab] or "surveys"[tiab] or "interview"[tiab] or "interviews"[tiab] or "health assessment*"[tiab] or inventory or inventories or measurement* or "sentinel surveillance"[mesh] or "public health surveillance"[mesh])))) and ("Non-alcoholic Fatty Liver Disease"[Mesh] OR "Nonalcoholic Fatty Liver"[tiab:~3] OR "Non alcoholic Fatty Liver"[tiab:~3] OR "Non-alcoholic Fatty Liver"[tiab:~3] OR "Nonalcoholic Steatohepatitis"[tiab:~3] OR "Nonalcoholic Steatohepatitides"[tiab:~3] OR "Non-alcoholic Steatohepatitis"[tiab:~3] OR "Non-alcoholic Steatohepatitides"[tiab:~3] OR "nonalcoholic liver steatosis"[tiab:~3] OR "non-alcoholic liver steatosis"[tiab:~3] OR "nonalcoholic hepatosteatosis"[tiab:~3] OR "non-alcoholic hepato-steatosis"[tiab:~3] OR "nonalcoholic liver FLD"[tiab:~3] OR "metabolic-associated fatty liver disease"[Mesh] OR "Metabolic associated fatty liver"[tiab:~3]" OR "metabolic-associated fatty liver"[tiab:~3]) NOT (("case reports"[publication type] or "comment"[publication type] or "editorial"[publication type] or "letter"[publication type] or "review"[publication type])) | 0 |
| --- | --- |

Obesity:

| ((((((("social determinants of health"[mesh] or "social risk"[tiab] or "social determinant"[tiab] or "social determinants"[tiab] or "socioeconomic determinant"[tiab] or "socioeconomic determinants"[tiab] or "health determinant"[tiab] or "socioeconomic factor"[tiab] or "socioeconomic factors"[tiab] or "health determinants"[tiab] or "health structural determinant"[tiab] or "health structural determinants"[tiab] or "economic stability"[mesh] or "economic stability"[tiab] or "education access and quality"[mesh] or "education access"[tiab] or "education quality"[tiab] or "health care access and quality"[mesh] or "health care access"[tiab] or "healthcare access"[tiab] or "health care quality"[tiab] or "healthcare quality"[tiab] or "neighborhood and built environment"[mesh] or "neighborhood environment"[tiab] or "built environment"[tiab] or "social and community context"[mesh] or "social context"[tiab] or "community context"[tiab])) and ("mass screening"[mesh:noexp] or "multiphasic screening"[mesh] or "health surveys"[mesh:noexp] or "screen*"[tiab] or "questionnaire"[tiab] or "questionnaires"[tiab] or "survey"[tiab] or "surveys"[tiab] or "interview"[tiab] or "interviews"[tiab] or "health assessment*"[tiab] or inventory or inventories or measurement* or "sentinel surveillance"[mesh] or "public health surveillance"[mesh])))) and ("anthropometry"[mesh] or "body mass index"[mesh] or "body weight"[mesh] or "overweight"[mesh] or "obesity"[mesh] or "life style"[mesh] or "adiposity"[mesh] or "metabolic syndrome"[mesh] or anthropometry[tiab] or "body mass"[tiab] or "body mass index"[tiab] or "body weight"[tiab] or overweight[tiab] or "over weight"[tiab] or obesity[tiab] or obese[tiab] or lifestyle[tiab] or "life style"[tiab] or adiposity[tiab] or "metabolic syndrome"[tiab])) not (("case reports"[publication type] or "comment"[publication type] or "editorial"[publication type] or "letter"[publication type] or "review"[publication type])) | 3,288 |
| --- | --- |

Hypertension:

| ((((((("social determinants of health"[mesh] or "social risk"[tiab] or "social determinant"[tiab] or "social determinants"[tiab] or "socioeconomic determinant"[tiab] or "socioeconomic determinants"[tiab] or "health determinant"[tiab] or "socioeconomic factor"[tiab] or "socioeconomic factors"[tiab] or "health determinants"[tiab] or "health structural determinant"[tiab] or "health structural determinants"[tiab] or "economic stability"[mesh] or "economic stability"[tiab] or "education access and quality"[mesh] or "education access"[tiab] or "education quality"[tiab] or "health care access and quality"[mesh] or "health care access"[tiab] or "healthcare access"[tiab] or "health care quality"[tiab] or "healthcare quality"[tiab] or "neighborhood and built environment"[mesh] or "neighborhood environment"[tiab] or "built environment"[tiab] or "social and community context"[mesh] or "social context"[tiab] or "community context"[tiab])) and ("mass screening"[mesh:noexp] or "multiphasic screening"[mesh] or "health surveys"[mesh:noexp] or "screen*"[tiab] or "questionnaire"[tiab] or "questionnaires"[tiab] or "survey"[tiab] or "surveys"[tiab] or "interview"[tiab] or "interviews"[tiab] or "health assessment*"[tiab] or inventory or inventories or measurement* or "sentinel surveillance"[mesh] or "public health surveillance"[mesh])))) AND (Hypertension[Mesh] OR hypertension[tiab] OR "blood pressure"[tiab] OR "blood pressures"[tiab] OR "arterial pressure"[tiab] "arterial pressures"[tiab] OR "systolic pressure"[tiab] OR "systolic pressures"[tiab] OR "diastolic pressure"[tiab] OR "diastolic pressure"[tiab] OR hypertensi*[tiab] OR prehypertens*[tiab] OR "pre hypertension"[tiab] OR "pre hypertensive"[tiab] OR "prehypertensive"[tiab]) NOT (("case reports"[publication type] or "comment"[publication type] or "editorial"[publication type] or "letter"[publication type] or "review"[publication type])) | 683 |
| --- | --- |

Diabetes:

| ((((((("social determinants of health"[mesh] or "social risk"[tiab] or "social determinant"[tiab] or "social determinants"[tiab] or "socioeconomic determinant"[tiab] or "socioeconomic determinants"[tiab] or "health determinant"[tiab] or "socioeconomic factor"[tiab] or "socioeconomic factors"[tiab] or "health determinants"[tiab] or "health structural determinant"[tiab] or "health structural determinants"[tiab] or "economic stability"[mesh] or "economic stability"[tiab] or "education access and quality"[mesh] or "education access"[tiab] or "education quality"[tiab] or "health care access and quality"[mesh] or "health care access"[tiab] or "healthcare access"[tiab] or "health care quality"[tiab] or "healthcare quality"[tiab] or "neighborhood and built environment"[mesh] or "neighborhood environment"[tiab] or "built environment"[tiab] or "social and community context"[mesh] or "social context"[tiab] or "community context"[tiab])) and ("mass screening"[mesh:noexp] or "multiphasic screening"[mesh] or "health surveys"[mesh:noexp] or "screen*"[tiab] or "questionnaire"[tiab] or "questionnaires"[tiab] or "survey"[tiab] or "surveys"[tiab] or "interview"[tiab] or "interviews"[tiab] or "health assessment*"[tiab] or inventory or inventories or measurement* or "sentinel surveillance"[mesh] or "public health surveillance"[mesh])))) AND (("Diabetes Mellitus, Type 2"[Mesh] OR "type 2 diabetes mellitus"[Mesh] OR "diabetes mellitus type 2"[Mesh] OR "Glucose Tolerance"[Mesh] OR "glucose tolerance"[All Fields] OR "impaired glucose tolerance"[All Fields] OR IGT OR "impaired fasting glucose" OR IFG OR "Glucose Intolerance"[MeSH] OR "glucose intolerance"[All Fields] OR "Prediabetic State"[MeSH] OR "prediabetic state"[All Fields] OR prediabet* OR "pre diabetes"[All Fields] OR "diabetes mellitus type 2"[All Fields] OR "type 2 diabetes mellitus"[All Fields]) OR ("Blood Glucose"[Mesh] OR "blood glucose"[tiab] OR "Glucose Tolerance Test"[Mesh] OR OGTT[tiab] OR "glucose tolerance test"[ti] OR "Glycated Hemoglobin A"[Mesh] OR "hemoglobin A1c" OR HbA1c OR "fasting plasma glucose"[tiab]) OR ("HbA(1c)"[tiab] or HbA1[tiab] or HbA1c[tiab] or "HbA 1c"[tiab])) NOT (("case reports"[publication type] or "comment"[publication type] or "editorial"[publication type] or "letter"[publication type] or "review"[publication type])) | 488 |
| --- | --- |

Hyperlipidemia:

| ((((("social determinants of health"[mesh] or "social risk"[tiab] or "social determinant"[tiab] or "social determinants"[tiab] or "socioeconomic determinant"[tiab] or "socioeconomic determinants"[tiab] or "health determinant"[tiab] or "socioeconomic factor"[tiab] or "socioeconomic factors"[tiab] or "health determinants"[tiab] or "health structural determinant"[tiab] or "health structural determinants"[tiab] or "economic stability"[mesh] or "economic stability"[tiab] or "education access and quality"[mesh] or "education access"[tiab] or "education quality"[tiab] or "health care access and quality"[mesh] or "health care access"[tiab] or "healthcare access"[tiab] or "health care quality"[tiab] or "healthcare quality"[tiab] or "neighborhood and built environment"[mesh] or "neighborhood environment"[tiab] or "built environment"[tiab] or "social and community context"[mesh] or "social context"[tiab] or "community context"[tiab])) and ("mass screening"[mesh:noexp] or "multiphasic screening"[mesh] or "health surveys"[mesh:noexp] or "screen*"[tiab] or "questionnaire"[tiab] or "questionnaires"[tiab] or "survey"[tiab] or "surveys"[tiab] or "interview"[tiab] or "interviews"[tiab] or "health assessment*"[tiab] or inventory or inventories or measurement* or "sentinel surveillance"[mesh] or "public health surveillance"[mesh])))) AND (Hyperlipidemia[Mesh] OR dyslipidemia[Mesh] OR hypertriglyceridemia[Mesh] OR "hyperlipidemia" OR "dyslipidemia" OR "hypertriglyceridemia" OR "high cholesterol" OR "high LDL" OR "low HDL" OR "lipid" OR "lipids") NOT (("case reports"[publication type] or "comment"[publication type] or "editorial"[publication type] or "letter"[publication type] or "review"[publication type])) | 197 |
| --- | --- |

**Embase:**

NAFLD:

| ('social determinants of health'/exp OR 'social determinants of health' OR 'social risk':ab,ti OR 'social determinant':ab,ti OR 'social determinants':ab,ti OR 'socioeconomic determinant':ab,ti OR 'socioeconomic determinants':ab,ti OR 'health determinant':ab,ti OR 'socioeconomic factor':ab,ti OR 'socioeconomic factors':ab,ti OR 'health determinants':ab,ti OR 'health structural determinant':ab,ti OR 'health structural determinants':ab,ti OR 'economic stability'/exp OR 'economic stability' OR 'economic stability':ab,ti OR 'education access and quality' OR 'education access':ab,ti OR 'education quality':ab,ti OR 'health care access and quality' OR 'health care access':ab,ti OR 'healthcare access':ab,ti OR 'health care quality':ab,ti OR 'healthcare quality':ab,ti OR 'neighborhood and built environment' OR 'neighborhood environment':ab,ti OR 'built environment':ab,ti OR 'social and community context' OR 'social context':ab,ti OR 'community context':ab,ti) AND ('mass screening'/exp OR 'multiphasic screening'/exp OR 'health surveys'/exp OR 'screen*':ab,ti OR 'questionnaire':ab,ti OR 'questionnaires':ab,ti OR 'survey':ab,ti OR 'surveys':ab,ti OR 'interview':ab,ti OR 'interviews':ab,ti OR 'health assessment*':ab,ti OR inventory:ab,ti OR inventories:ab,ti OR measurement*:ab,ti OR 'sentinel surveillance'/exp OR 'public health surveillance'/exp) AND ('nonalcoholic fatty liver'/exp OR 'nonalcoholic steatohepatitis'/exp OR 'nonalcoholic fatty liver':ab,ti OR 'nonalcoholic steatohepatitis':ab,ti OR 'metabolic fatty liver':ab,ti OR 'nonalcoholic liver steatosis':ab,ti OR 'nonalcoholic liver steatosis'/exp OR 'nonalcoholic liver fld':ab,ti OR 'metabolic associated fatty liver':ab,ti OR 'metabolic-associated fatty liver':ab,ti) | 34 |
| --- | --- |

Obesity:

| ('social determinants of health'/exp OR 'social risk':ab,ti OR 'social determinant':ab,ti OR 'social determinants':ab,ti OR 'socioeconomic determinant':ab,ti OR 'socioeconomic determinants':ab,ti OR 'health determinant':ab,ti OR 'socioeconomic factor':ab,ti OR 'socioeconomic factors':ab,ti OR 'health determinants':ab,ti OR 'health structural determinant':ab,ti OR 'health structural determinants':ab,ti OR 'economic stability'/exp OR 'economic stability':ab,ti OR 'education access and quality' OR 'education access':ab,ti OR 'education quality':ab,ti OR 'health care access and quality' OR 'health care access':ab,ti OR 'healthcare access':ab,ti OR 'health care quality':ab,ti OR 'healthcare quality':ab,ti OR 'neighborhood and built environment' OR 'neighborhood environment':ab,ti OR 'built environment':ab,ti OR 'social and community context' OR 'social context':ab,ti OR 'community context':ab,ti) AND ('mass screening'/exp OR 'multiphasic screening'/exp OR 'health surveys'/exp OR 'screen*':ab,ti OR 'questionnaire':ab,ti OR 'questionnaires':ab,ti OR 'survey':ab,ti OR 'surveys':ab,ti OR 'interview':ab,ti OR 'interviews':ab,ti OR 'health assessment*':ab,ti OR inventory:ab,ti OR inventories:ab,ti OR measurement*:ab,ti OR 'sentinel surveillance'/exp OR 'public health surveillance'/exp) AND (“'anthropometry'/exp OR 'anthropometry' OR 'body mass index'/exp OR 'body mass index' OR 'body weight'/exp OR 'body weight' OR 'overweight'/exp OR 'overweight' OR 'obesity'/exp OR 'obesity' OR 'life style'/exp OR 'life style' OR 'adiposity'/exp OR 'adiposity' OR 'metabolic syndrome'/exp OR 'metabolic syndrome' OR anthropometry:ab,ti OR 'body mass':ab,ti OR 'body mass index':ab,ti OR 'body weight':ab,ti OR overweight:ab,ti OR 'over weight':ab,ti OR obesity:ab,ti OR obese:ab,ti OR lifestyle:ab,ti OR 'life style':ab,ti OR adiposity:ab,ti OR 'metabolic syndrome':ab,ti”) | 4,285 |
| --- | --- |

Hypertension:

| ('social determinants of health'/exp OR 'social risk':ab,ti OR 'social determinant':ab,ti OR 'social determinants':ab,ti OR 'socioeconomic determinant':ab,ti OR 'socioeconomic determinants':ab,ti OR 'health determinant':ab,ti OR 'socioeconomic factor':ab,ti OR 'socioeconomic factors':ab,ti OR 'health determinants':ab,ti OR 'health structural determinant':ab,ti OR 'health structural determinants':ab,ti OR 'economic stability'/exp OR 'economic stability':ab,ti OR 'education access and quality' OR 'education access':ab,ti OR 'education quality':ab,ti OR 'health care access and quality' OR 'health care access':ab,ti OR 'healthcare access':ab,ti OR 'health care quality':ab,ti OR 'healthcare quality':ab,ti OR 'neighborhood and built environment' OR 'neighborhood environment':ab,ti OR 'built environment':ab,ti OR 'social and community context' OR 'social context':ab,ti OR 'community context':ab,ti) AND ('mass screening'/exp OR 'multiphasic screening'/exp OR 'health surveys'/exp OR 'screen*':ab,ti OR 'questionnaire':ab,ti OR 'questionnaires':ab,ti OR 'survey':ab,ti OR 'surveys':ab,ti OR 'interview':ab,ti OR 'interviews':ab,ti OR 'health assessment*':ab,ti OR inventory:ab,ti OR inventories:ab,ti OR measurement*:ab,ti OR 'sentinel surveillance'/exp OR 'public health surveillance'/exp) AND ('hypertension'/exp OR hypertension:ab,ti OR 'blood pressure':ab,ti OR 'blood pressures':ab,ti OR 'arterial pressure':ab,ti) AND 'arterial pressures':ab,ti OR 'systolic pressure':ab,ti OR 'systolic pressures':ab,ti OR 'diastolic pressure':ab,ti OR hypertensi*:ab,ti OR prehypertens*:ab,ti OR 'pre hypertension':ab,ti OR 'pre hypertensive':ab,ti OR 'prehypertensive':ab,ti) | 954 |
| --- | --- |

Diabetes:

| ('social determinants of health'/exp OR 'social risk':ab,ti OR 'social determinant':ab,ti OR 'social determinants':ab,ti OR 'socioeconomic determinant':ab,ti OR 'socioeconomic determinants':ab,ti OR 'health determinant':ab,ti OR 'socioeconomic factor':ab,ti OR 'socioeconomic factors':ab,ti OR 'health determinants':ab,ti OR 'health structural determinant':ab,ti OR 'health structural determinants':ab,ti OR 'economic stability'/exp OR 'economic stability':ab,ti OR 'education access and quality' OR 'education access':ab,ti OR 'education quality':ab,ti OR 'health care access and quality' OR 'health care access':ab,ti OR 'healthcare access':ab,ti OR 'health care quality':ab,ti OR 'healthcare quality':ab,ti OR 'neighborhood and built environment' OR 'neighborhood environment':ab,ti OR 'built environment':ab,ti OR 'social and community context' OR 'social context':ab,ti OR 'community context':ab,ti) AND ('mass screening'/exp OR 'multiphasic screening'/exp OR 'health surveys'/exp OR 'screen*':ab,ti OR 'questionnaire':ab,ti OR 'questionnaires':ab,ti OR 'survey':ab,ti OR 'surveys':ab,ti OR 'interview':ab,ti OR 'interviews':ab,ti OR 'health assessment*':ab,ti OR inventory:ab,ti OR inventories:ab,ti OR measurement*:ab,ti OR 'sentinel surveillance'/exp OR 'public health surveillance'/exp) AND ('diabetes mellitus, type 2'/exp OR 'type 2 diabetes mellitus'/exp OR 'diabetes mellitus type 2'/exp OR 'glucose tolerance'/exp OR 'glucose tolerance':ab,ti OR 'impaired glucose tolerance':ab,ti OR igt OR 'impaired fasting glucose' OR ifg OR 'glucose intolerance'/exp OR 'glucose intolerance':ab,ti OR 'prediabetic state'/exp OR 'prediabetic state':ab,ti OR prediabet* OR 'pre diabetes':ab,ti OR 'diabetes mellitus type 2':ab,ti OR 'type 2 diabetes mellitus':ab,ti) | 468 |
| --- | --- |

Hyperlipidemia:

| ('social determinants of health'/exp OR 'social risk':ab,ti OR 'social determinant':ab,ti OR 'social determinants':ab,ti OR 'socioeconomic determinant':ab,ti OR 'socioeconomic determinants':ab,ti OR 'health determinant':ab,ti OR 'socioeconomic factor':ab,ti OR 'socioeconomic factors':ab,ti OR 'health determinants':ab,ti OR 'health structural determinant':ab,ti OR 'health structural determinants':ab,ti OR 'economic stability'/exp OR 'economic stability':ab,ti OR 'education access and quality' OR 'education access':ab,ti OR 'education quality':ab,ti OR 'health care access and quality' OR 'health care access':ab,ti OR 'healthcare access':ab,ti OR 'health care quality':ab,ti OR 'healthcare quality':ab,ti OR 'neighborhood and built environment' OR 'neighborhood environment':ab,ti OR 'built environment':ab,ti OR 'social and community context' OR 'social context':ab,ti OR 'community context':ab,ti) AND ('mass screening'/exp OR 'multiphasic screening'/exp OR 'health surveys'/exp OR 'screen*':ab,ti OR 'questionnaire':ab,ti OR 'questionnaires':ab,ti OR 'survey':ab,ti OR 'surveys':ab,ti OR 'interview':ab,ti OR 'interviews':ab,ti OR 'health assessment*':ab,ti OR inventory:ab,ti OR inventories:ab,ti OR measurement*:ab,ti OR 'sentinel surveillance'/exp OR 'public health surveillance'/exp) AND ('hyperlipidemia'/exp OR 'dyslipidemia'/exp OR 'hypertriglyceridemia'/exp OR 'hyperlipidemia':ab,ti OR 'dyslipidemia':ab,ti OR 'hypertriglyceridemia':ab,ti OR 'high cholesterol' OR 'high ldl' OR 'low hdl' OR 'lipid' OR 'lipids') | 417 |
| --- | --- |

**CINAHL COMPLETE:**

NAFLD:

| ((MH “social determinants of health") OR (TI ("social risk" OR “social determinant” OR “social determinants” OR “socioeconomic determinant” OR “socioeconomic determinants” OR “health determinant” OR “health determinants” OR “socioeconomic factor” OR “socioeconomic factors” OR “health structural determinant” OR “health structural determinants” OR “economic stability” OR “education access and quality” OR “education access” OR “education quality” OR “health care access and quality” OR “health care access” OR “healthcare access” OR “health care quality” OR “healthcare quality” OR “neighborhood and built environment” OR “neighborhood environment” OR “built environment” OR “social and community context” OR “social context” OR “community context”)) OR (AB ("social risk" OR “social determinant” OR “social determinants” OR “socioeconomic determinant” OR “socioeconomic determinants” OR “health determinant” OR “health determinants” OR “socioeconomic factor” OR “socioeconomic factors” OR “health structural determinant” OR “health structural determinants” OR “economic stability” OR “education access and quality” OR “education access” OR “education quality” OR “health care access and quality” OR “health care access” OR “healthcare access” OR “health care quality” OR “healthcare quality” OR “neighborhood and built environment” OR “neighborhood environment” OR “built environment” OR “social and community context” OR “social context” OR “community context”)) OR (MH "economic stability”) OR (MH "neighborhood and built environment”) OR (MH “education access and quality”) OR (MH “health care access and quality”) OR (MH "social and community context")) AND ((MH "mass screening") OR (MH "multiphasic screening”) OR (MH "health surveys”) OR (MH “sentinel surveillance”) OR (MH “public health surveillance”) OR (TI (“screen*” OR “questionnaire” OR “questionnaires” OR “survey” OR “surveys” OR “interview” OR “interviews” OR “health assessment*” OR “inventory” OR “inventories” OR “measurement”)) OR (AB (“screen*” OR “questionnaire” OR “questionnaires” OR “survey” OR “surveys” OR “interview” OR “interviews” OR “health assessment*” OR “inventory” OR “inventories” OR “measurement”)) AND ((MH "non- alcoholic fatty liver disease") OR (MH "nonalcoholic fatty liver") OR (MH “nonalcoholic liver steatosis”) OR (MH “metabolic-associated fatty liver disease”) OR (TI ("non-alcoholic fatty liver" OR “nonalcoholic steatohepatitis” OR “nonalcoholic steatohepatitides” OR “non-alcoholic steatohepatitis” OR “non- alcoholic steatohepatitides” OR “nonalcoholic liver steatosis” OR “non- alcoholic liver steatosis” OR “nonalcoholic hepatosteatosis” OR “non- alcoholic hepato- steatosis” OR “nonalcoholic liver FLD” OR “metabolic associated fatty liver” OR “metabolic- associated fatty liver”)) OR (AB ("non-alcoholic fatty liver" OR “nonalcoholic steatohepatitis” OR “nonalcoholic steatohepatitides” OR “non-alcoholic steatohepatitis” OR “non- alcoholic steatohepatitides” OR “nonalcoholic liver steatosis” OR “non- alcoholic liver steatosis” OR “nonalcoholic hepatosteatosis” OR “non- alcoholic hepato- steatosis” OR “nonalcoholic liver FLD” OR “metabolic associated fatty liver” OR “metabolic- associated fatty liver”)) | 0 |
| --- | --- |

Obesity:

| ((MH “social determinants of health") OR (TI ("social risk" OR “social determinant” OR “social determinants” OR “socioeconomic determinant” OR “socioeconomic determinants” OR “health determinant” OR “health determinants” OR “socioeconomic factor” OR “socioeconomic factors” OR “health structural determinant” OR “health structural determinants” OR “economic stability” OR “education access and quality” OR “education access” OR “education quality” OR “health care access and quality” OR “health care access” OR “healthcare access” OR “health care quality” OR “healthcare quality” OR “neighborhood and built environment” OR “neighborhood environment” OR “built environment” OR “social and community context” OR “social context” OR “community context”)) OR (AB ("social risk" OR “social determinant” OR “social determinants” OR “socioeconomic determinant” OR “socioeconomic determinants” OR “health determinant” OR “health determinants” OR “socioeconomic factor” OR “socioeconomic factors” OR “health structural determinant” OR “health structural determinants” OR “economic stability” OR “education access and quality” OR “education access” OR “education quality” OR “health care access and quality” OR “health care access” OR “healthcare access” OR “health care quality” OR “healthcare quality” OR “neighborhood and built environment” OR “neighborhood environment” OR “built environment” OR “social and community context” OR “social context” OR “community context”)) OR (MH "economic stability”) OR (MH "neighborhood and built environment”) OR (MH “education access and quality”) OR (MH “health care access and quality”) OR (MH "social and community context")) AND ((MH "mass screening") OR (MH "multiphasic screening”) OR (MH "health surveys”) OR (MH “sentinel surveillance”) OR (MH “public health surveillance”) OR (TI (“screen*” OR “questionnaire” OR “questionnaires” OR “survey” OR “surveys” OR “interview” OR “interviews” OR “health assessment*” OR “inventory” OR “inventories” OR “measurement”)) OR (AB (“screen*” OR “questionnaire” OR “questionnaires” OR “survey” OR “surveys” OR “interview” OR “interviews” OR “health assessment*” OR “inventory” OR “inventories” OR “measurement”)) AND ((MH "anthropometry") OR (MH "body mass index") OR (MH "body weight") OR (MH "overweight”) OR (MH "obesity") OR (MH "life style") OR (MH "adiposity") OR (MH "metabolic syndrome") OR (TI (“anthropometry” OR “body mass” OR “body mass index” OR “body weight” OR overweight OR “over weight” OR obesity OR obese OR lifestyle OR “life style” OR adiposity OR “metabolic syndrome”)) OR (AB (“anthropometry” OR “body mass” OR “body mass index” OR “body weight” OR overweight OR “over weight” OR obesity OR obese OR lifestyle OR “life style” OR adiposity OR “metabolic syndrome”)) | 1,307 |
| --- | --- |

Hypertension:

| ((MH “social determinants of health") OR (TI ("social risk" OR “social determinant” OR “social determinants” OR “socioeconomic determinant” OR “socioeconomic determinants” OR “health determinant” OR “health determinants” OR “socioeconomic factor” OR “socioeconomic factors” OR “health structural determinant” OR “health structural determinants” OR “economic stability” OR “education access and quality” OR “education access” OR “education quality” OR “health care access and quality” OR “health care access” OR “healthcare access” OR “health care quality” OR “healthcare quality” OR “neighborhood and built environment” OR “neighborhood environment” OR “built environment” OR “social and community context” OR “social context” OR “community context”)) OR (AB ("social risk" OR “social determinant” OR “social determinants” OR “socioeconomic determinant” OR “socioeconomic determinants” OR “health determinant” OR “health determinants” OR “socioeconomic factor” OR “socioeconomic factors” OR “health structural determinant” OR “health structural determinants” OR “economic stability” OR “education access and quality” OR “education access” OR “education quality” OR “health care access and quality” OR “health care access” OR “healthcare access” OR “health care quality” OR “healthcare quality” OR “neighborhood and built environment” OR “neighborhood environment” OR “built environment” OR “social and community context” OR “social context” OR “community context”)) OR (MH "economic stability”) OR (MH "neighborhood and built environment”) OR (MH “education access and quality”) OR (MH “health care access and quality”) OR (MH "social and community context")) AND ((MH "mass screening") OR (MH "multiphasic screening”) OR (MH "health surveys”) OR (MH “sentinel surveillance”) OR (MH “public health surveillance”) OR (TI (“screen*” OR “questionnaire” OR “questionnaires” OR “survey” OR “surveys” OR “interview” OR “interviews” OR “health assessment*” OR “inventory” OR “inventories” OR “measurement”)) OR (AB (“screen*” OR “questionnaire” OR “questionnaires” OR “survey” OR “surveys” OR “interview” OR “interviews” OR “health assessment*” OR “inventory” OR “inventories” OR “measurement”)) AND ((MH “hypertension”) OR (TI (“hypertensi*” OR “blood pressure” OR “blood pressures” OR “arterial pressure” OR “arterial pressures” OR “systolic pressure” OR “systolic pressures” OR “diastolic pressure” OR “diastolic pressures” OR “prehypertension” OR “pre hypertension” OR “pre hypertensive” OR “prehypertensive”)) OR (AB (“hypertensi*” OR “blood pressure” OR “blood pressures” OR “arterial pressure” OR “arterial pressures” OR “systolic pressure” OR “systolic pressures” OR “diastolic pressure” OR “diastolic pressures” OR “prehypertension” OR “pre hypertension” OR “pre hypertensive” OR “prehypertensive”)) | 392 |
| --- | --- |

Diabetes:

| ((MH “social determinants of health") OR (TI ("social risk" OR “social determinant” OR “social determinants” OR “socioeconomic determinant” OR “socioeconomic determinants” OR “health determinant” OR “health determinants” OR “socioeconomic factor” OR “socioeconomic factors” OR “health structural determinant” OR “health structural determinants” OR “economic stability” OR “education access and quality” OR “education access” OR “education quality” OR “health care access and quality” OR “health care access” OR “healthcare access” OR “health care quality” OR “healthcare quality” OR “neighborhood and built environment” OR “neighborhood environment” OR “built environment” OR “social and community context” OR “social context” OR “community context”)) OR (AB ("social risk" OR “social determinant” OR “social determinants” OR “socioeconomic determinant” OR “socioeconomic determinants” OR “health determinant” OR “health determinants” OR “socioeconomic factor” OR “socioeconomic factors” OR “health structural determinant” OR “health structural determinants” OR “economic stability” OR “education access and quality” OR “education access” OR “education quality” OR “health care access and quality” OR “health care access” OR “healthcare access” OR “health care quality” OR “healthcare quality” OR “neighborhood and built environment” OR “neighborhood environment” OR “built environment” OR “social and community context” OR “social context” OR “community context”)) OR (MH "economic stability”) OR (MH "neighborhood and built environment”) OR (MH “education access and quality”) OR (MH “health care access and quality”) OR (MH "social and community context")) AND ((MH "mass screening") OR (MH "multiphasic screening”) OR (MH "health surveys”) OR (MH “sentinel surveillance”) OR (MH “public health surveillance”) OR (TI (“screen*” OR “questionnaire” OR “questionnaires” OR “survey” OR “surveys” OR “interview” OR “interviews” OR “health assessment*” OR “inventory” OR “inventories” OR “measurement”)) OR (AB (“screen*” OR “questionnaire” OR “questionnaires” OR “survey” OR “surveys” OR “interview” OR “interviews” OR “health assessment*” OR “inventory” OR “inventories” OR “measurement”)) AND ((MH “diabetes Mellitus, type 2”) OR (MH “type 2 diabetes mellitus”) OR (MH “diabetes mellitus type 2”) OR (MH “Glucose Tolerance”) OR (MH “glucose intolerance”) OR (MH “prediabetic state”) OR (TI (“glucose tolerance” OR “impaired glucose tolerance” OR “IGT” OR “impaired fasting glucose” OR “IFG” or “glucose intolerance” OR “prediabetic state” OR “prediabet*” OR “pre diabetes” OR “diabetes mellitus type 2” OR “type 2 diabetes mellitus”)) OR (AB (“glucose tolerance” OR “impaired glucose tolerance” OR “IGT” OR “impaired fasting glucose” OR “IFG” or “glucose intolerance” OR “prediabetic state” OR “prediabet*” OR “pre diabetes” OR “diabetes mellitus type 2” OR “type 2 diabetes mellitus”)) | 172 |
| --- | --- |

Hyperlipidemia:

| ((MH “social determinants of health") OR (TI ("social risk" OR “social determinant” OR “social determinants” OR “socioeconomic determinant” OR “socioeconomic determinants” OR “health determinant” OR “health determinants” OR “socioeconomic factor” OR “socioeconomic factors” OR “health structural determinant” OR “health structural determinants” OR “economic stability” OR “education access and quality” OR “education access” OR “education quality” OR “health care access and quality” OR “health care access” OR “healthcare access” OR “health care quality” OR “healthcare quality” OR “neighborhood and built environment” OR “neighborhood environment” OR “built environment” OR “social and community context” OR “social context” OR “community context”)) OR (AB ("social risk" OR “social determinant” OR “social determinants” OR “socioeconomic determinant” OR “socioeconomic determinants” OR “health determinant” OR “health determinants” OR “socioeconomic factor” OR “socioeconomic factors” OR “health structural determinant” OR “health structural determinants” OR “economic stability” OR “education access and quality” OR “education access” OR “education quality” OR “health care access and quality” OR “health care access” OR “healthcare access” OR “health care quality” OR “healthcare quality” OR “neighborhood and built environment” OR “neighborhood environment” OR “built environment” OR “social and community context” OR “social context” OR “community context”)) OR (MH "economic stability”) OR (MH "neighborhood and built environment”) OR (MH “education access and quality”) OR (MH “health care access and quality”) OR (MH "social and community context")) AND ((MH "mass screening") OR (MH "multiphasic screening”) OR (MH "health surveys”) OR (MH “sentinel surveillance”) OR (MH “public health surveillance”) OR (TI (“screen*” OR “questionnaire” OR “questionnaires” OR “survey” OR “surveys” OR “interview” OR “interviews” OR “health assessment*” OR “inventory” OR “inventories” OR “measurement”)) OR (AB (“screen*” OR “questionnaire” OR “questionnaires” OR “survey” OR “surveys” OR “interview” OR “interviews” OR “health assessment*” OR “inventory” OR “inventories” OR “measurement”)) AND ((MH “hyperlipidemia”) OR (MH “dyslipidemia”) OR (MH “hypertriglyceridemia”) OR (TI (“hyperlipidemia” OR “dyslipidemia” OR “hyptriglyceridemia” OR “high cholesterol” OR “high LDL” OR “low HDL” OR “lipid” OR “lipids”)) OR (AB (“hyperlipidemia” OR “dyslipidemia” OR “hyptriglyceridemia” OR “high cholesterol” OR “high LDL” OR “low HDL” OR “lipid” OR “lipids”)) | 79 |
| --- | --- |
